# Translating a rodent measure of negative bias into humans: the impact of induced anxiety and unmedicated mood and anxiety disorders

**DOI:** 10.1017/S0033291718004117

**Published:** 2019-01-26

**Authors:** Jessica Aylward, Claire Hales, Emma Robinson, Oliver J. Robinson

**Affiliations:** 1 Neuroscience and Mental Health Group, Institute of Cognitive Neuroscience, 17–19 Queen Square, University College London, WC1N 3AZ, London, UK; 2 School of Physiology and Pharmacology, Biomedical Sciences Building, University Walk, University of Bristol, BS8 1TD, Bristol, UK

**Keywords:** Affective bias, anxiety, back-translation, computational psychiatry, depression, drift diffusion model

## Abstract

**Background:**

Mood and anxiety disorders are ubiquitous but current treatment options are ineffective for many sufferers. Moreover, a number of promising pre-clinical interventions have failed to translate into clinical efficacy in humans. Improved treatments are unlikely without better animal–human translational pipelines. Here, we translate a rodent measure of negative affective bias into humans, exploring its relationship with (1) pathological mood and anxiety symptoms and (2) transient induced anxiety.

**Methods:**

Adult participants (age = 29 ± 11) who met criteria for mood or anxiety disorder symptomatology according to a face-to-face neuropsychiatric interview were included in the symptomatic group. Study 1 included *N* = 77 (47 = asymptomatic [female = 21]; 30 = symptomatic [female = 25]), study 2 included *N* = 47 asymptomatic participants (25 = female). Outcome measures were choice ratios, reaction times and parameters recovered from a computational model of reaction time – the drift diffusion model (DDM) – from a two-alternative-forced-choice task in which ambiguous and unambiguous auditory stimuli were paired with high and low rewards.

**Results:**

Both groups showed over 93% accuracy on unambiguous tones indicating intact discrimination, but symptomatic individuals demonstrated increased negative affective bias on ambiguous tones [proportion high reward = 0.42 (s.d. = 0.14)] relative to asymptomatic individuals [0.53 (s.d. = 0.17)] as well as a significantly reduced DDM drift rate. No significant effects were observed for the within-subjects anxiety-induction.

**Conclusions:**

Humans with pathological anxiety symptoms directly mimic rodents undergoing anxiogenic manipulation. The lack of sensitivity to transient anxiety suggests the paradigm might be more sensitive to clinically relevant symptoms. Our results establish a direct translational pipeline (and candidate therapeutics screen) from negative affective bias in rodents to pathological mood and anxiety symptoms in humans.

## Introduction

Mood and anxiety disorders are extremely prevalent worldwide, with huge psychological, economical and social costs (Beddington *et al*., 2008). ‘Affective biases’, which span many domains of cognition, are core features of these disorders (MacLeod *et al*., 1986). For example, anxious and depressed individuals demonstrate increased sensitivity to aversive stimuli (Mogg and Bradley, 2006), an attentional bias towards threatening information (MacLeod *et al*., 1986), and biased interpretation of ambiguous information (Hirsch and Mathews, 1997) [for a review see Roiser *et al*. (2012)]. These biases both precipitate the onset of disorders and contribute to their maintenance (Kendler *et al*., 2004; Harmer *et al*., 2009; Roiser *et al*., 2012). Targeting these biases is therefore a key goal of treatment development.

Unfortunately, for a sizeable number of individuals, current treatments do not lead to clinical improvement (Joffe *et al*., 1996; Psychological Therapies: Annual report on the use of IAPT services Psychological Therapies: Annual Report on the use of IAPT services, England, 2015–16, 2016). Recent years have moreover seen a number of high-profile failures in drug development (Choi *et al*., 2014; Scannell *et al*., 2016). Among the reasons for this, is that some pre-clinical animal tests do not adequately translate the human behaviour they are designed to model (Choi *et al*., 2014; Badre *et al*., 2015; Scannell *et al*., 2016). Indeed there are no tasks that are identical across species; some prominent examples – the forced swim test (Porsolt *et al*., 1977), or tail suspension test (Steru *et al*., 1985) – do not have clear human analogues. We argue, therefore, that developing *identical* paradigms across humans and animal models will help reduce pre-clinical to clinical translation failure. Instead of *scaling-back* paradigms developed in humans into animals, the present paper takes a paradigm developed within the constraints of an animal model, and directly translates it for human use.

Specifically, we translate a rodent model of affective bias into humans. In the animal task (Hales *et al*., 2016), rats learn to correctly respond to high- or low-frequency tones, which are associated (100%) with high or low rewards (food pellets). In the test-phase they also respond to an ambiguous mid-tone randomly reinforced with both high/low reward outcomes. The optimal response to this ambiguous stimulus, which is exactly equidistant between the unambiguous stimuli, and is reinforced with 50% of each outcome, is to press the high reward button with a probability of 0.5. However, a ‘pessimistic’ response is to more frequently assume that this ambiguous stimulus will lead to the less good outcome, and hence press the low reward button more than 50% of the time. This would result in a probability pressing the high reward button of less than 0.5, and hence represent negative affective bias. Rats administered an anxiogenic drug or subjected to chronic stress (repeated restraint stress and social isolation) (Hales *et al*., 2016) display increased negative affective bias in choice behaviour. No significant behavioural effect is observed for rats undergoing acute stress (restraint) manipulation.

Here, we explored the impact of two types of anxiety on a human version of this task: (a) pathological anxiety in mood and anxiety disorders, and (b) acute stress induced using threat of unpredictable shock. The latter stress induction is a well-validated and reliable technique, also translated from animal models (Robinson *et al*., 2011; Aylward and Robinson, 2017). Critically, it allows the interaction between cognition and anxiety to be explored within-subjects. It elicits ‘adaptive anxiety’ responses such as response inhibition and harm avoidance (Boureau and Dayan, 2011; Robinson *et al*., 2013*a*; Aylward and Robinson, 2017) as well as ‘negative bias’ (Robinson *et al*., 2011, 2012, 2013*b*) in healthy individuals. A related, albeit more complex, version of the present task has previously been tested in healthy participants. Participants responded to a tone paired with reward (to obtain money) and a tone paired with punishment (to avoid punishment). In a test phase participants made more avoidance responses to an ambiguous tone, demonstrating a bias towards avoiding punishment – i.e. an avoidance bias (Anderson *et al*., 2012). Notably, this avoidance bias in responding was correlated negatively with a self-reported *state* anxiety level. As such, we predicted that on our novel, directly translated rodent task, induced and pathological anxiety would be associated with a negative affective bias.

Computational models can make specific predictions about the underlying mechanisms that drive behaviour and enable a more fine-grained view of decision-making and how it changes in pathological states (Robinson and Chase, 2017). One such model – the drift diffusion model (DDM) – has been applied to rodent data on this task (Hales *et al*., 2016). This model parameterises decision-making as a process of noisy accumulation of evidence (Ratcliff *et al*., 2016) and is able to accurately model the reaction times to stimuli on two-alternative forced choice tasks. Negative bias following acute pharmacological manipulation and chronic stress in rats was accompanied by increased ‘boundary separation’ parameters (more information required in order to reach a decision), whereas reduced ‘drift rate’ (rate of information accumulation) parameters were seen following the pharmacological manipulation. In this paper we applied both the EZ drift model (Wagenmakers *et al*., 2007) – a pared down version of the DDM (van Ravenzwaaij *et al*., 2016) as well as a full Bayesian hierarchical DDM (Wiecki *et al*., 2013) to our human data.

We therefore tested two predictions. Firstly, considering the well-documented biases in pathological anxiety (MacLeod and Mathews, 2012) and prior work with related tasks (Anderson *et al*., 2012), we predicted that individuals with mood and anxiety disorders, relative to the asymptomatic group, would demonstrate increased negative affective bias in this task. Secondly, as induced anxiety instantiates biases across cognition (Robinson *et al*., 2013*b*), we predicted that in asymptomatic individuals, threat of shock would also instantiate a negative affective bias. In both cases, we predicted that negative bias in choice behaviour would be associated with alterations to drift diffusion parameters.

## Method

### Participants

Participants were recruited using internet advertisements and via subject databases held at University College London. The only group difference in recruitment was the wording of the advertisements; asymptomatic healthy participants replied to advertisements asking for participants with no psychiatric symptoms; whilst participants with low mood and/or anxiety symptoms replied to advertisements asking for participants who self-defined as experiencing persistent low mood/anxiety symptoms.

A total of 77 participants were included in study 1: 47 asymptomatic participants (mean age = 28.83, s.d. = 10.52; 25 female) and 30 (*N* = 31 originally, but one excluded as they failed to follow task instructions), unmedicated participants with low mood and/or anxiety symptoms (mean age = 28.93, s.d. = 10.92; 21 female). A total of 47 asymptomatic participants were included in study 2 (mean age = 28.96, s.d. = 10.45; 25 female; 46 overlap with study 1). The neutral version of the task (study 1) was always completed first to ensure consistency with the symptomatic group (who did not complete the stress version). Participants could be aged between 18 and 65 years.

### Symptomatic group details

The symptomatic group comprised individuals who met criteria for mood and anxiety disorders. As depressive and anxiety symptoms are highly comorbid and may not have distinct underlying causes, we include a mixed sample in our symptomatic group (see Table 1 and online Supplement). Following an initial screening process, participants who met criteria for mood or anxiety disorder symptomatology according to a face-to-face Mini International Neuropsychiatric Interview [M.I.N.I. (Lecrubier *et al*., 1997)] were included in the symptomatic group, those who did not meet any (past/present) criteria according to the M.I.N.I. were included in the asymptomatic group. The State-Trait Anxiety Inventory [STAI (Spielberger *et al*., 1983)] was also collected, as well as additional measures (see Table 1 for full details). Exclusion criteria are listed in the online Supplement.

**Table 1.** Demographic and Clinical information

Demographic	HC	ANX
Age	28.83 (10.52)	28.93 (10.92)
Female	55.32%	70%
Caucasian	48%	56.67%
Education	16.68 (2.23)	14.37 (5.42)
STAI state	29.94 (8.06)	51.83 (9.82)
STAI trait	33.72 (10.12)	59.43 (10.05)
BDI	3.30 (3.78)	25.10 (10.36)
Ham-D	–	16.14 (5.52)
Raven's	8.96 (2.13)	8.21 (2.74)

HC, asymptomatic healthy control; ANX, symptomatic individual; STAI, State-Trait Anxiety Inventory; BDI, Beck depression Inventory; Ham-D, Hamilton depression inventory; Ravens's, Raven's progressive matrices.

## Procedure

Participants provided written informed consent to take part (ethical approval from UCL ethics reference: 6198/001 and 1764/001). They completed a task coded using the Cogent (Wellcome Trust Centre for Neuroimaging and Institute of Cognitive Neuroscience, UCL, London, UK) toolbox for Matlab (2014b, The MathWorks, Inc., Natick, MA, United States). Scripts are available at: 10.6084/m9.figshare.4868303.

### Acquisition Phase

**Fig. 1.**
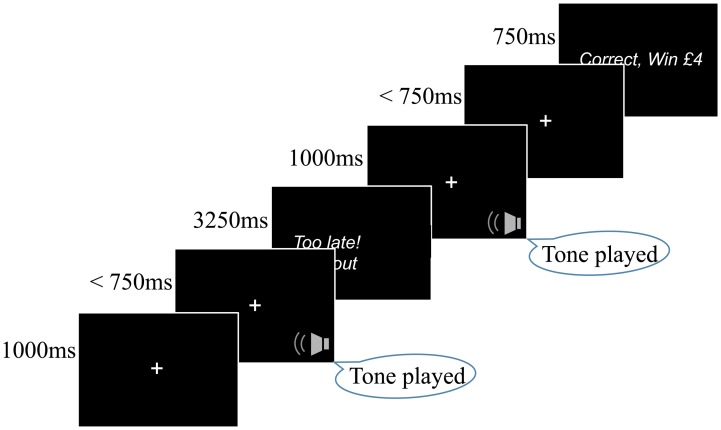
Participants were required to make a key press (‘z’ or ‘m’ key) following a tone played for 1000 ms. After making their response, participants received feedback on their performance. Correct responses saw feedback appear on the screen for 750 ms, whilst incorrect responses, or responses made outside the 750 ms window, saw feedback appear on the screen for 3250 ms. The task consisted of 120 trials, during which 40 low (500 Hz), mid-tone (750 Hz) and high (1000 Hz) tones were presented. High-/low-frequency tones were 100% associated with wins of £1 or £4 (contingency counterbalanced across participants). Note the order of trials and outcomes is for illustration purposes only.

A task schematic is presented in Fig. 1. During the acquisition block, participants heard high (1000 Hz) and low tones (500 Hz), these frequencies were lower than the rat task to account for cross-species differences in hearing. The two tones were associated with different reward values (tone/reward pairings were counterbalanced across participants). They were instructed to learn to make correct key presses following each tone (‘z’ or ‘m’ key on a laptop keyboard) and informed that correct responses would be rewarded. They were told that they should try and maximise earnings. Ten low and ten high tones, randomly presented, were played during the practice block. A tone was played for 1000 ms followed by an inter stimulus interval of 750 ms. A white fixation cross appeared in the middle of the screen during this time. Participants could make their response from the onset of the tone presentation. Following the key press feedback was provided. ‘Correct, Win £1’ appeared for 750 ms following a correct response to the low reward tone (low/high frequency, counterbalanced). ‘Correct, Win £4’ appeared for 750 ms following a correct response to the high reward tone (low/high frequency, counterbalanced). ‘Timeout for incorrect response’ appeared for 3250 ms following an incorrect or slow response. This delay was provided to match directly with the rodent version of the task (Hales *et al*., 2016). The acquisition block enabled participants to understand the key/tone pairings (counterbalanced across participants). The practice block could last between 50 and 100 s.

### Testing phase

The tone/reward pairings remained the same as in acquisition but the participants were also presented with a mid-point, ambiguous tone (750 Hz) which fell directly in between the low and high tones. Participants were informed that they might hear other tones and that if the tone was unclear, that they should make a key press that corresponded to the closest tone. For half of the trials this mid-tone was associated with a high reward outcome, and for the other half of the trials it was associated with a low reward outcome (with feedback contingent on whether participants happened to select this random outcome). The sequence of mid-tone outcomes was created uniquely for each individual (a list of alternating outcomes was sorted using the MATLAB randperm function). As in the practice block, a tone was played for 1000 ms, followed by an interstimulus interval of 750 ms. Participants made their response as quickly as possible following the tone presentation. Following correct responses the feedback was presented on the screen for 750 ms, whilst following incorrect or slow responses ‘Timeout for incorrect response’ was presented on the screen for 3250 ms.

### Study 1: symptomatic group *v*. asymptomatic controls

#### Stimuli Details

The main task consisted of 120 trials (40 low/mid/high tones, randomly presented). The main task could therefore last between 300 and 600 s.

### Study 2: induced anxiety version

#### Shock work-up

A Digitimer DS5 Constant Current Stimulator (Digitimer Ltd., Welwyn Garden City, UK) delivered the shocks, via two electrodes attached to the participant's non-dominant wrist. The shock intensity was increased until the subjective rating was ‘unpleasant, but not painful’ (Schmitz and Grillon, 2012).

#### Stimuli details

**Fig. 2.**
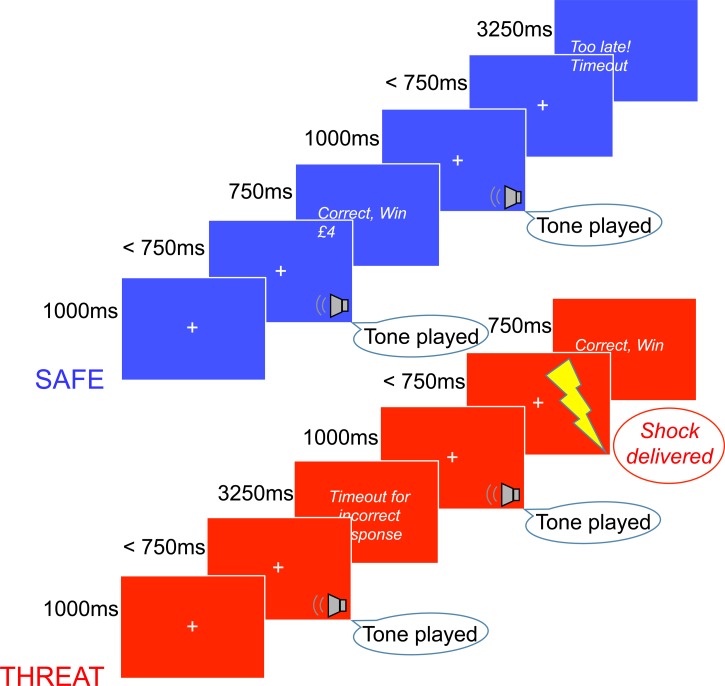
Participants were required to make a key press (‘z’/’m’) following a tone played for 1000 ms. After making their response, participants received feedback on their performance. Feedback for correct responses lasted 750 ms, whilst feedback for incorrect (or slower than 750 ms) responses lasted 3250 ms. During the safe condition, in which the background was blue, participants were not at risk of shock. During the threat condition, in which the background was red, participants were at risk of unpredictable electric shock. Low (500 Hz), mid-tone (750 Hz) and high (1000 Hz) tones were presented. High/low tones were 100% associated with wins of £1 or £4 (contingency counterbalanced across participants).

A task schematic is presented in Fig. 2. The task was performed under instructed threat and safe conditions in the same manner as Aylward and Robinson (2017). Participants were told that they would be at risk of an unpredictable shock (independent of their behavioural response), during a threat block (red background). Participants were told that they would be free from shock during a safe block (blue background). The order of the conditions (threat or safe first) was counterbalanced across participants. Colours were not counterbalanced as prior work has shown this effect to be independent of background colour (Grillon *et al*., 1993, 2006). Each block (total = 4) consisted of 60 randomly presented trials (20 low/mid/high tones; total = 240). The maintenance task could therefore last between 600 and 1200 s. Participants either received a shock in the first threat block (post-threat-trial = 45), in the second threat block (post-threat-trial = 96) or at both of these times (randomised across participants). As a manipulation check, participants retrospectively rated their anxiety (out of 10) under threat and safe conditions.

### Statistical analyses

Reaction time (RT) and bias measures (data available at: 10.6084/m9.figshare.4868303) were analysed using SPSS Version 22 (IBM Crop, Armonk, NY). For all analyses, *p* = 0.05, was considered significant. Affective bias (percentage of ambiguous tones classified as high reward) was calculated by dividing the number of ‘high reward’ responses made to the mid-tone by the total number of key presses made to the mid-tone (note that the pairing of the high/low *frequencies* with high/low reward was counterbalanced across participants, so this refers to the probability of selecting the button associated with high *reward*, not high frequency) and compared across groups or conditions using paired sample *t* tests and Bayesian equivalents. RT to respond to the mid-tone was normally distributed and was analysed using independent and paired sample *t* tests for studies 1 and 2 respectively.

Bayesian statistics were run [JASP, version 0.7 (JASP, 2016)], employing the default prior. The Bayesian approach considers the likelihood of the data if the alternative hypothesis is true versus if the null hypothesis is true, allowing for inferences to be made about which model best explains the data. Bayesian analysis of variances and *t* tests were used to generate BF_10_ factors which provided evidence for a model of interest relative to a null model. A model with a BF_10_ > 1 signifies that model is better at explaining the data relative to the null model, and vice versa for BF_10_ < 1. To interpret the magnitude differences between models the following labels were assigned to BF_10_: anecdotal (1–3), substantial (3–10), strong (10–30) decisive (>100) (Jeffreys, 1998).

Mean RT, variance and proportion of positive responses to the mid-tone were also fed into the EZ-DM (script available at: 10.6084/m9.figshare.4868303). The parameters of interest were: boundary separation (*a*), drift rate (*v*) and non-decision time (*t*). These refer to the amount of information required before a response can be made (*a*), the rate at which this information is accumulated (*v*) and the proportion of the RT that is not accounted for by evidence accumulation (*t*).

Finally, EZ-DM analyses were supplemented by full hierarchical Bayesian model comparison using the Hierarchical Bayesian estimation of the Drift-Diffusion Model in Python (HDDM) toolbox (Wiecki *et al*., 2013). The modelled parameters were identical to the above, but this approach also enabled the inclusion of a bias parameter (*z*), which denotes the starting point between the boundaries. The data for all trials were included in this analysis (stratified into ambiguous mid tone and unambiguous high/low reward trial types) and parameters fit using an Markov Chain Monte Carlo (MCMC) sampling approach implemented using PyMC (Patil *et al*., 2010) (2000 MCMC samples with a burn-in of 20 samples; all winning models obtained Gelman-Rubin statistics ~1). The influence of adding and subtracting parameters was examined by comparing deviance information criterion (DIC) scores across models. The most extreme 5% of RTs was automatically excluded from all model fitting by the toolbox [assuming that outliers come from a uniform distribution; see Wiecki *et al*. (2013) for more details] to account for lapses and facilitate model fitting. Follow-up analysis on recovered parameters was run in a comparable manner to the EZ diffusion analysis and supplemented with a full Bayesian model comparison approach in which the impact of including group or condition in the hierarchical model was tested, and the posteriors of parameters that depended on additional hierarchy plotted for models achieving or exceeding parity of model fits with the basic model. The winning models showed good parameter recovery on posterior predictive checks.

Correlation analyses were also run to investigate correlations between STAI trait anxiety scores, affective bias and drift rate.

## Results

### Study 1

#### Choice behaviour

**Fig. 3.**
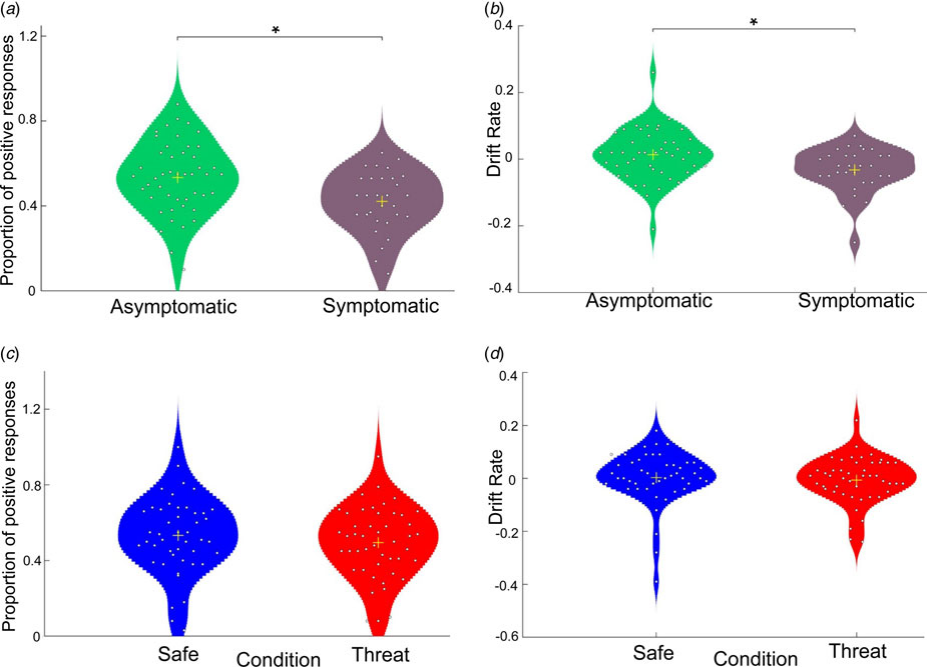
The impact of pathological and induced anxiety on ambiguous mid-tone predictions. Violin plots of the proportion of positive responses made to ambiguous tone and EZDM ‘drift rate’ – the rate of accumulation of evidence to classify a tone as high reward (shaded area represents a smoothed histogram; yellow cross represents the mean; each circle represents an individual). (a) Symptomatic individuals had more negative bias (*p* = 0.003, BF_10_ = 12.51) and (b) a more negative drift rate towards classifying the mid-tone as high reward (*p* = 0.008, BF_10_ = 5.22). However, there was (c) no significant difference in affective bias following induced anxiety (*p* = 0.06, BF_10_ = 0.863) and (d) no significant difference in drift rate across conditions (*p* > 0.125, BF_10_ < 1). EZDM, ‘easy’ diffusion model; BF, Bayes factor.

High reward and low reward tone accuracy was high (Table 2) and comparable across groups (*t*_(75)_ = 0.96, *p* = 0.338, *d* = 0.22, and *t*_(75)_ = 0.28, *p* = 0.78, *d* = −0.06, respectively; no trial × group interaction in accuracy *F*_(1,75)_ = 1.7, *p* = 0.2 or RT *F*_(1,75)_ = 0.2, *p* = 0.8). However, there was a significant effect of group on mid-tone choice (*t*_(75)_ = 3.08, *p* = 0.003, *d* = 0.732, see Fig. 3). The symptomatic group was less likely to associate the mid-tone with high reward compared to the asymptomatic group. Bayesian analysis provided strong evidence for a significant difference in affective bias between groups (BF_10_ = 12.51). Subjects were also significantly slower on these mid-tone trials than both high (*t*_(76)_ = 11.8, *p* < 0.001) and low (*t*_(76)_ = 15.1, *p* = 0.003) reward trials.

**Table 2.** Average choice, accuracy and reaction time (ms) to all tones in study 1

Asymptomatic	Accuracy (s.d.)
Low reward tone	0.98 (0.05)
High reward tone	0.93 (0.083)
Symptomatic	Accuracy
Low reward tone	0.97 (0.039)
High reward tone	0.95 (0.069)
Group	Proportion high reward responses to mid-tone
Asymptomatic	0.53 (0.17)
Symptomatic	0.42 (0.14)
Asymptomatic	Reaction time
Low reward tone	819.51 (212.00)
Mid-tone	942.41 (181.78)
High reward tone	757.33 (228.47)
Symptomatic	Reaction time
Low reward tone	763.17 (197.78)
Mid-tone	894.54 (203.57)
High reward tone	694.56 (194.32)

#### Reaction time

See Table 2 for average RT to all tone types across group. Time to respond to the mid-tone did not differ across groups (*t*_(75)_ = 1.08, *p* = 0.29, *d* = 0.248). Bayesian analysis favoured the null model (BF_10_ = 0.40).

#### DDM

##### EZ-DM

Despite comparable overall RTs there was a significant difference in drift rate between groups (*t*_(75)_ = 2.70, *p* = 0.008); but not boundary separation (*t*_(75)_ = −0.79, *p* = 0.43) or non-decision time (*t*_(75)_ = 1.3, *p* = 0.96). The symptomatic group had a slower drift rate towards making a positive choice to the mid-tone (asymptomatic mean = 0.013, s.d. = 0.075, symptomatic mean = −0.032, s.d. = 0.066; see Fig. 3). Bayesian analysis provided substantial evidence for a difference between groups in drift rate (BF_10_ = 5.22; all other BF_10_ < 0.31).

##### HDDM

**Fig. 4.**
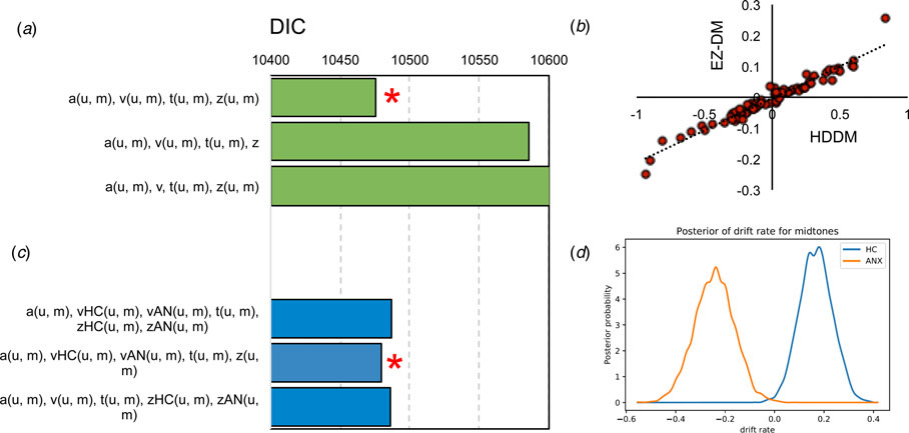
Hierarchical drift diffusion modelling of pathological anxiety reveals (a) a winning model (*) that includes separate drift rate (*v*), boundary separation (*a*) non-decision time (*t*) and bias (*z*) parameters for unambiguous (u) and ambiguous mid-tone (m) trial types based on lowest DIC scores. The *v* parameters recovered using this approach (HDDM) (b) correlate tightly with those recovered from the EZ-DM model. Including group in the model fitting procedure (c) demonstrates that the best model (*) fits the *v* parameter alone separately across groups. This is because, as can be seen on the posterior recovered samples, the (d) *v* parameter was more negative in patients than controls. HC, asymptomatic healthy control; ANX, symptomatic individual.

A wide model search was completed (see online Supplement) across a range of parameters and within-subject factors. The three best models are presented in Fig. 4*a*. The winning model comprised a model with drift rate, boundary separation, bias and non-decision time parameters (fitted separately across ambiguous mid tone and unambiguous trial types). As with the EZ-DM model, parameters extracted from this winning model demonstrated significant difference in ambiguous mid-tone drift rate between groups (*t*_(75)_ = 3.0, *p* = 0.004); but not boundary separation (*t*_(75)_ = −1.2, *p* = 0.22), non-decision time (*t*_(75)_ = 1.4, *p* = 0.15) or bias (*t*_(75)_ = −1.4, *p* = 0.89). The winning model parameters showed a tight correspondence (all *r* > 0.8, *p* < 0.001) with the EZ-DM parameters (see drift rate; Fig. 4*b*). However, one advantage of the full hierarchical approach is that we can include group in the model fitting procedure. This approach revealed a winning model (of equivalent fit to the model fit across groups) where the drift rate parameter alone is separated by group. Posterior distributions demonstrate that this is because *v* on mid tones is lower in patients relative to controls (Fig. 4*d*). In short, the full hierarchical model is consistent with the basic EZ-DM model.

### Correlations

There was a strong positive correlation between affective bias and both drift rate measures (*r* > 0.98, *p* < 0.001), those who had a bias away from choosing high rewards had a slower drift rate towards high rewards.

There was weak evidence for a correlation between affective bias and STAI trait scores (*r* = *−*0.207, *p*(*two-tailed*) = 0.07, *p*(*one-tailed*) = 0.035) as well as weak evidence for a correlation between drift rate and STAI trait scores (EZ-DM *r* = −0.21, *p*(*two-tailed*) = 0.066, *p*(*one-tailed*) = 0.033; HDDM *r* = −0.22, *p*(*two-tailed*) = 0.053, *p*(*one-tailed*) = 0.027). In other words higher anxiety was associated with a reduced drift rate to the high reward choice. Additional exploratory correlations can be found in the online Supplement.

### Study 2

#### Threat of shock manipulation check

Participant anxiety ratings were significantly higher during the threat condition relative to the safe condition *t*_(44)_ = 8.92, *p* < 0.001, *d* = 1.88 (safe mean = 1.64, s.d. = 1.05; threat mean = 4.93, s.d. = 2.21). Bayesian analysis provided decisive evidence that a model with a main effect of threat was the winning model (BF_10_ = 4.68 × 10^8^).

#### Choice behaviour

Accuracy for the high reward and low reward tones were high (Table 3) and comparable across conditions (*t*_(46)_ = 0.975, *p* = 0.335, *d* = 0.02, and *t*_(46)_ = 1.597, *p* = 0.117, *d* = 0.33, respectively). During the threat condition the proportion of mid-tones associated with high reward was smaller relative to the safe condition but did not achieve significance (*t*_(46)_ = 1.93, *p* = 0.06, *d* = −0.28; see Fig. 3). Bayesian analysis anecdotally favoured a model with a main effect of condition (BF_10_ = 1.019). Subjects were also significantly slower on mid-tone trials than both high (*t*_(46)_ = 10.3, *p* < 0.001) and low (*t*_(46)_ = 11.3, *p* < 0.001) reward trials. There was no interaction in mid-tone choice behaviour between the condition and number of shocks (*F*_(1,45)_ < 0.001, *p* = 0.98) nor between condition and the time of the first shock (*F*_(3,43)_ = 0.34, *p* = 0.80).

**Table 3.** Average choice, accuracy, and reaction time (ms) to respond to tones in each condition in study 2

Tone/condition	Accuracy (s.d.)
Low reward tone (safe)	0.99 (0.030)
High reward tone (safe)	0.96 (0.047)
Low reward tone (threat)	0.98 (0.036)
High reward tone (threat)	0.95 (0.061)
Condition	Proportion high reward responses to mid-tone
Safe	0.53 (0.20)
Threat	0.49 (0.19)
Tone/condition	Reaction time
Low reward tone (safe)	815.29 (192.09)
Mid-tone (safe)	954.36 (185.63)
High reward tone (safe)	830.46 (185.60)
Low reward tone (threat)	767.84 (206.84)
Mid-tone (threat)	970.05 (168.97)
High reward tone (threat)	787.37 (205.78)

#### Reaction time

See Table 3 for RT to different tone types across conditions. There was no difference between conditions in time taken to respond to the mid-tone (*t*_(46)_ = 1.24, *p* = 0.221, *d* = 0.26). Bayesian analysis confirmed that the null model was the winning model (BF_10_ = 0.325).

#### DDM

##### EZ-DM

There was no significant difference between conditions in drift rate, non-decision time or boundary separation in decision-making to the mid-tones (*p*s > 0.125). Bayesian analysis confirmed that the null model was the winning model in all cases (BF_10_ < 1).

##### HDDM

**Fig. 5.**
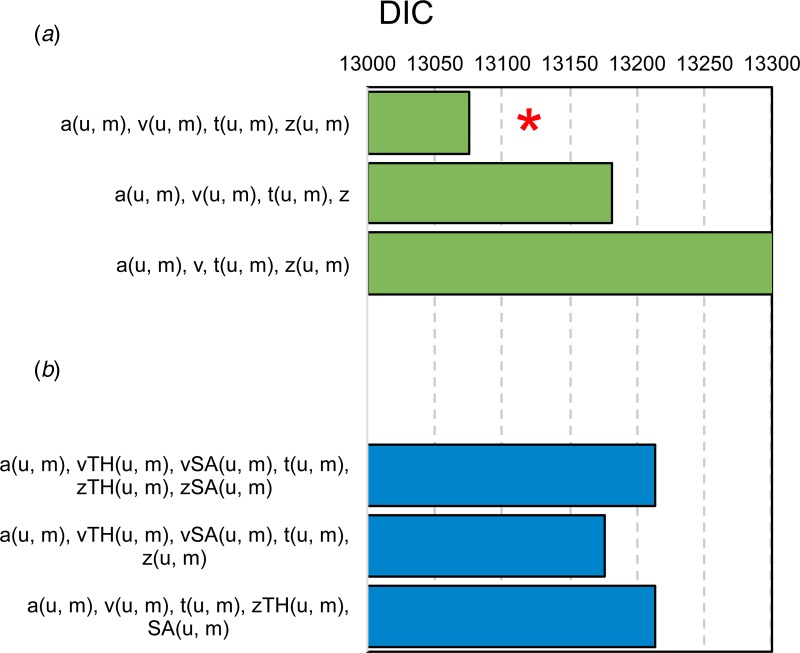
Hierarchical drift diffusion modelling of induced anxiety reveals (a) a winning model (*) that includes separate drift rate (*v*), boundary separation (*a*) non-decision time (*t*) and bias (*z*) parameters unambiguous (u) and ambiguous mid-tone (m) trial types based on lowest DIC scores. Including condition in the model fitting procedure (b) provides substantially worse fits, thereby providing no evidence for an effect of condition.

The winning model comprised drift rate, boundary separation, non-decision time and bias parameter all fitted separately across ambiguous and unambiguous trials (Fig. 5*a*). This was the same model as study 1; however, this time, adding condition (Fig. 5*b*) into the hierarchy in this winning model resulted in substantially worse fits, thereby providing no justification for dividing trials by condition. In other words, the full hierarchical procedure again agreed with the EZ-DM procedure.

## Conclusion

In this study we directly translate a rodent measure of affective bias. We demonstrate that pathological mood and anxiety disorders, but not transient-induced anxiety in asymptomatic individuals, are associated with increased negative affective bias in task performance. This bias can, moreover, be attributed to reduced ‘drift rate’ on a computational model of reaction times.

**Fig. 6.**
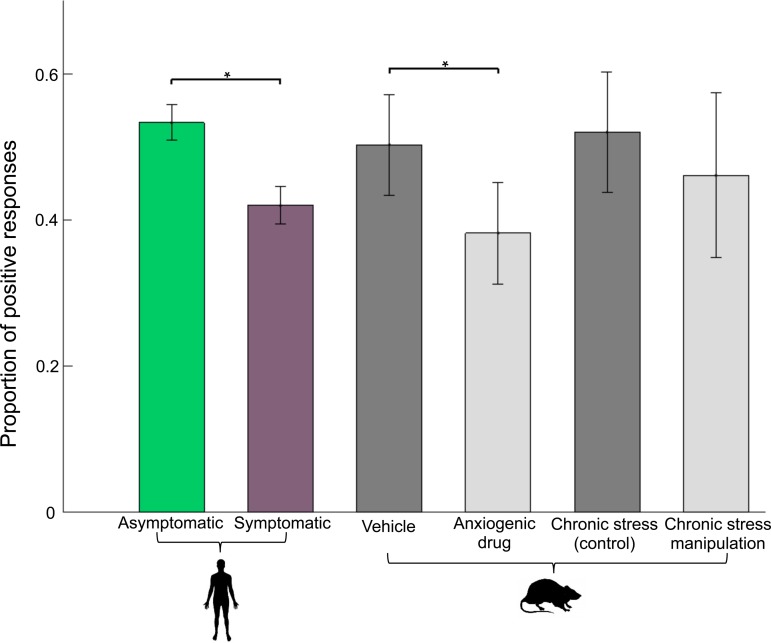
Cross-species performance comparison. Plots illustrating the overlap of human pathological anxiety and rodent anxiety models on choice performance (*p < 0.05). Data presented in (Hales *et al*., 2016). After acute pharmacological manipulation with FG7142 (3 or 5 mg; average dose plotted), rats showed an increased negative affective bias in choice behaviour on the ambiguous tone, relative to vehicle. For the chronic stress manipulation between weeks 3 and 4 (post-stress intervention average of 6 post-stress intervention weeks plotted), rats showed an increased negative affective bias in choice behaviour on the ambiguous tone, relative to control.

Our results demonstrate that individuals with mood and anxiety disorders are more likely to interpret an ambiguous stimulus in a pessimistic light; i.e. assume that it is more likely to lead to the worse of two potential outcomes. As such they align with evidence documenting negative affective bias in mood and anxiety disorders (Hirsch and Mathews, 1997; Anderson *et al*., 2012; Mathews, 2012) as well as two prior (conceptually different) studies (White *et al*., 2010; Dillon *et al*., 2015) linking mood disorder symptomatology to drift rates on the DDM. Critically, the anxiety-negative bias interaction translates the impact of (a) acute anxiogenic pharmacological manipulation and (b) chronic stress in the rodent task (Hales *et al*., 2016) (Fig. 6) into humans, suggesting that these rodent manipulations may be suitable preclinical screens for candidate therapeutics.

Threat of shock instantiates negative affective biases across many areas of cognition (Robinson *et al*., 2013*b*), but counter to predictions, induced anxiety in asymptomatic individuals did not reliably shift performance on this task. One potential explanation is that, in the asymptomatic group, the induced anxiety task was always completed following the neutral version of the task. This may have increased familiarity with the task and counteracted any biases. However, it is also worth noting that the observation that decision-making is more sensitive to pathological than transient anxiety is also consistent with chronic *v*. acute restraint stress in rats (Hales *et al*., 2016). Perhaps, therefore, acute environmental anxiety promotes *adaptive* harm-avoidance (Robinson *et al*., 2013*b*), by increasing attentional and perceptual biases towards threats, without influencing higher-order decision-making processes. Supporting this is evidence demonstrating that, whilst encoding of values in ‘lower-level’ brain valuation structures changes as a function of threat-induced anxiety, decision-making behaviour remains unperturbed (Engelmann *et al*., 2015; Robinson *et al*., 2015; Charpentier *et al*., 2016) by threat of shock. It could therefore be that lower-level learning and memory are *immediately* influenced by transient states, but that the impact upon higher order processes builds up over time (Anderson *et al*., 2013). If correct, this suggests that, at least on the present measure, there is something quantifiably different between transient anxiety in healthy humans and pathological anxiety. From a clinical perspective this is unsurprising, but it is notable because some effects do overlap across induced and pathological anxiety (Robinson *et al*., 2013*b*, 2014; Robinson and Chase, 2017). Finally, it is worth acknowledging that we may simply be underpowered to detect an effect of threat, perhaps because the manipulation was not strong enough. This is arguably unlikely considering increased anxiety ratings under threat, and the wide-ranging influence of induced anxiety on cognition (Robinson *et al*., 2013*b*). However, if correct it would mean that the within-subject effect of transient anxiety is considerably smaller than the between-subject effect detected in the group study.

Modifying affective biases in mood and anxiety disorders is crucial given their proposed role in the development and maintenance of symptoms (Kendler *et al*., 2004; Harmer *et al*., 2009; Roiser *et al*., 2012). Both pharmacological and psychological treatments (Dimidjian *et al*., 2006; Zarate *et al*., 2006; Fournier *et al*., 2010), are thought to exert their effects via altering affective biases (Roiser *et al*., 2012). In rodents, for instance, a similar task has been shown to be sensitive to anxiolytic manipulations; a positive bias is exhibited after treatment with the antidepressant venlafaxine (Hinchcliffe *et al*., 2017). Confirming the same effect on this task in a medicated human sample would therefore enhance the predictive validity of this task for drug testing new anxiolytics. It will also be important to confirm that the bias effects we see in our symptomatic group extend to treatment-seeking samples recruited through clinical services who may have more severe symptoms.

In addition to facilitating screening of novel anxiolytics, the present translational pipeline provides a potential means of understanding the mechanisms underpinning this negative bias (Stuart *et al*., 2015). Running causal studies in rodents can help us delineate the neurobiological processes underpinning biased choices on this task (Badre *et al*., 2015). Moreover, linking task performance to a formal model of decision-making (DDM) provides a step towards bridging the gap between brain and behaviour. Notably, in the rodent model that most closely mimics the choice behaviour of anxious humans (Hales *et al*., 2016), as well as in humans with anxiety disorders, the drift rate parameter was reduced. This suggests that, in both cases, anxiety reduces the rate of evidence accumulation (although it should be noted that, unlike the rodent mode, in humans the bias parameter was unaffected). Crucially, the parameters of this model are thought to be biophysically plausible; they can be computed by populations of neurons (Ratcliff and McKoon, 2008); taking us closer to being able to link underlying neural activity to psychiatric symptoms. Such links are necessary for a full mechanistic account of psychiatric symptoms and are the guiding principal of the burgeoning field of computational psychiatry (Huys *et al*., 2016).

It is worth noting, however, that the model in some ways recapitulates the model free analysis, in that a drift rate of zero indicates no bias and positive and negative drift rates indicate a bias towards the larger or smaller reward respectively. However, this affective bias could instead be driven by a change in the starting point (*z* otherwise known, not uncoincidentally, as the *bias* parameter), but inclusion of this parameter was not favoured in the full hierarchical modelling. In practical terms it is the specifics of the RT distributions (and their comparisons) that allow us to discriminate between these two possibilities (Ratcliff *et al*., 2016) so the modelling takes into account more of the information obtained from each individual (i.e. RT as well as choice probability), and ultimately enables us to make more precise predictions about the underlying mechanisms. Thus it is not that, when an anxious participant decides to select the lower reward button, they are a priori favoured to choose that option (i.e. *z*/bias); rather it is during the subsequent decision making phase that the ‘race’ to choose (i.e. accumulate evidence in favour of) the less favoured option is won (i.e. a negative drift rate). This also indicates that the effect is not due to learning – i.e. the participants have not learnt a prior – although future work is needed to clarify this.

Ultimately, we argue that improved treatments are unlikely without a better understanding of the underlying biological mechanisms that any putative treatments are attempting to target. Given the huge costs of mood and anxiety disorders; as well as the large number of individuals for whom none of our current treatments work; new and improved treatments, and therefore better methods of screening for such treatments, are long overdue. We propose that the task presented here may hold promise as a means of better screening for candidate treatments across humans and animal models.

## Supplementary material

For supplementary material accompanying this paper visit https://doi.org/10.1017/S0033291718004117. click here to view supplementary material
