# Supplementary material for: Translating a rodent measure of negative bias into humans: the impact of induced anxiety and unmedicated mood and anxiety disorders
Source: Psychol Med. 2019 Jan 26;50(2):237–46. doi: 10.1017/S0033291718004117 (PMC7083556; doi:10.1017/S0033291718004117)
Supplement: Supplementary file 1 [file S0033291718004117sup001.docx]

Supplementary Information

**Participants**

The sample included N = 15 individuals who met criteria for major depressive disorder (MDD; of these N=1 was experiencing symptoms but just missed criteria, N = 1 with a history of panic attacks, N = 1 for panic disorder lifetime, and N = 1 for past PTSD and Anorexia Nervosa with Binging), N = 11 met for both MDD and generalised anxiety disorder (GAD; of these N = 1 with panic symptoms, N = 2 with panic disorder lifetime, N = 1 with panic disorder current, N = 1 panic disorder current and PTSD), and N = 4 participants met criteria for GAD without MDD (of these N = 1 panic disorder lifetime, N = 1 past MDD and panic disorder lifetime, N = 1 past MDD, panic disorder current). All participants were unmedicated, but N = 5 were receiving current psychological treatment and included as they were still symptomatic. The average number of depressive episodes was 5 months (SD = 8.30), and mean current episode duration was 6 months (SD = 12.12).

**Flow chart**

REDCap Questionnaire Completed

Potential participants contacted by the researcher and a phone screening is conducted to investigate current low mood / anxiety, drug use and GP registration. Eligible participants invited to take part in the testing session.

*Exclusions:*

*Those who were not symptomatic, who had used drugs in the last 4 weeks, psychiatric medication in the last 6 months, and who were not registered with a GP were excluded.*

Mini-International Neuropsychiatric Interview (M.I.N.I; Sheehan et al., 1998) was administered to determine group status and eligibility.

Eligible ANX participants also completed the Hamilton Depression Rating Scale (Ham-D; Hamilton, 1960).

All participants completed the BDI (BDI; Beck et al., 1996), the State-Trait Anxiety Inventory (STAI; Spielberger, 1983), and the Raven’s Advanced Progressive Matrices (RAPM, 12-item short form; Arthur & Day, 1994).

*Exclusions:*

*ANX participants who met criteria for mania, hypomania or psychotic disorder. A first degree relative with bipolar disorder or schizophrenia, current/past neurological disorder, current/past learning disability, recreational drug use in the last month or past drug dependence (‘mild’ accepted if within an episode), and current/past alcohol dependence (‘mild’ accepted if within a depressive episode), psychiatric medication within last 6 months.*

*HC participants who met criteria for any MINI criteria, past or present, or a family history of depression / anxiety were excluded.*

Phone screening

Testing session

**Exclusion criteria**

Exclusion criteria included any psychiatric medication in the last 6 months, meeting M.I.N.I criteria for mania, hypomania, or psychotic disorder, having a first degree relative with bipolar disorder or schizophrenia, current or past neurological disorder, current/past learning disability, recreational drug use in the last month or past drug dependence (‘mild’ accepted if within an episode), and current/past alcohol dependence (‘mild’ accepted if within a depressive episode). Five participants were consuming alcohol three or more times per week, but were included as they did not meet criteria for current dependence. All participants were asked to abstain from alcohol for 24 hours prior to testing.

**HDDM initial models**

**
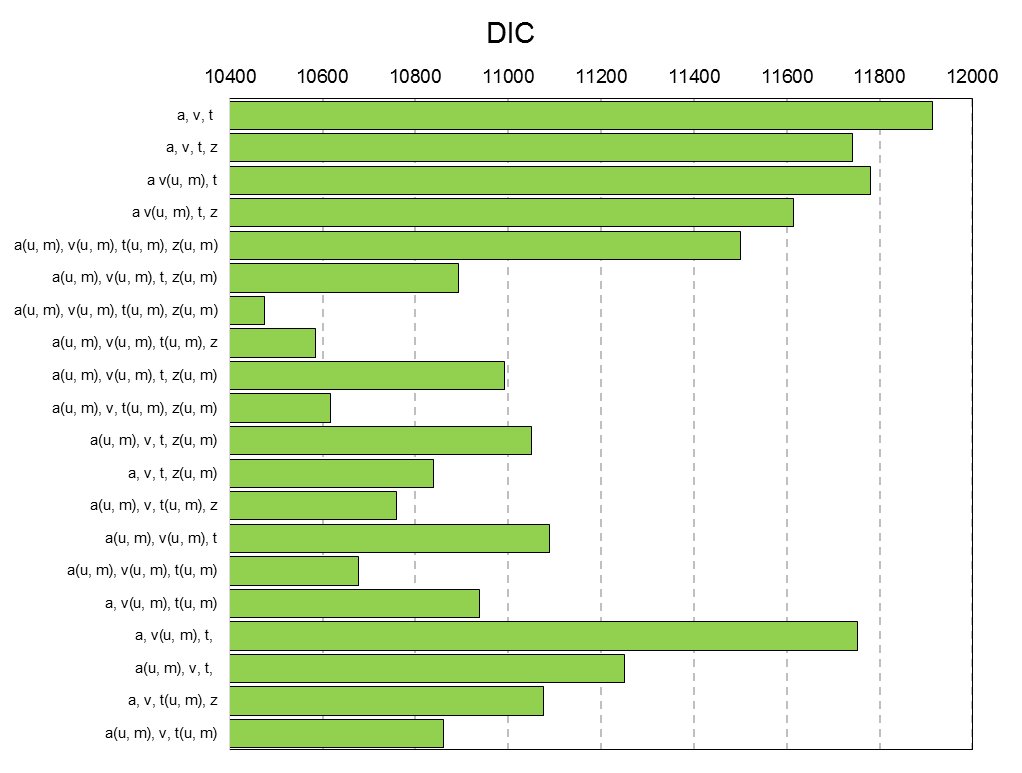
**

***Figure S1 DIC scores*** *for models with combinations of drift rate (v), boundary separation (a) non-decision time (t) and bias parameters separated by unambiguous (u) and ambiguous mid-tone (m) trial types fit across the 77 participants in study 1.*

**Exploratory analyses**

We looked at the influence of trait anxiety on performance in induced anxiety task (study 2). There was no significant interaction between anxiety and bias F(1,22) = 0.93, *p* = 0.57, *d=*0.27. Bayesian analysis confirmed that a model including a main effect of condition was the winning model (BF_10_=1.01). In a subset of participants (scoring in the Upper Quartile on STAI) there was no significant effect of condition (*t*_(11)=_0.179, *p=0.86, d*= 0.07). Bayesian analysis confirmed that a null model was the winning model (BF_10_ = 0.367).

To explore the specificity of the findings we ran exploratory analyses to examine any differences between anxious and depressed participants. We did not find any significant differences between symptoms F(2,29) = 0.331, *p* = 0.721) and Bayesian analysis confirmed that the null model was the winning model (BF_10_ = 0.278).

BDI and STAI scores explained a significant amount of the variance in the proportion of high reward responses made to the ambiguous tone (F(2,26) = 6.17, *p =* 0.003, *R = 0.38, R^2^ = 0.14*).

Including years of musical training as a covariate in a regression alongside group did not substantially change inference about the group effect (*t*_(74)_=3.03,p*=*0.003), suggesting that group effects are not driven by differences in the ability to discriminate tones (and years of training did not differ across groups (*t*_(75)_=-0.5,p*=*0.62).
